# Laparoscopic intrapancreatic accessory splenectomy: A case report of recurrent immune thrombocytopenia in a 33 years old male patient after 6 years of splenectomy

**DOI:** 10.1016/j.ijscr.2019.06.021

**Published:** 2019-06-16

**Authors:** Abdullah AlShammari, Dana Kalagi, Talal Hijji, Mohammad Aburahmah

**Affiliations:** a College of Medicine, Alfaisal University, P.O. Box 50927, Riyadh 11533, Saudi Arabia; b King Faisal Specialist Hospital and Research Center (KFSH&RC), P. O. Box 3354, Riyadh 11211, Saudi Arabia

**Keywords:** Laparoscopy, Splenectomy, Accessory spleen, Idiopatic thrombocytopenic purpura

## Abstract

**Introduction:**

Intra-pancreatic Accessory Spleen (IPAS) is a rare benign abnormality of splenic embryology that should be considered in any patient with recurrence of immune thrombocytopenia (ITP). The case demonstrates that remission could be achieved if an IPAS is correctly identified and safely removed.

**Case presentation:**

We report a 33-years-old male patient who presented with chronic ITP relapsed 3 years post initial splenectomy. The patient presented with severe thrombocytopenia requiring steroids and intravenous immunoglobulin (IVIG).

**Discussion:**

IPAS can be identified using enhanced CT scan along with Tc-99 m sulfur colloid scintigraphy. Our patient underwent CT scan of abdomen and pelvis and was found to have an IPAS originating from the tail of the pancreas which was subsequently removed laparoscopically. The patient was then tapered off steroids and was followed up regularly and found to have acceptable platelet levels and no symptoms for 30 months post-surgery.

**Conclusion:**

The diagnosis of IPAS can be non-invasively made by an experienced radiologist with the recent advancement of medical imaging. Recognizing IPAS as a possible cause of ITP recurrence, and using minimally invasive approach can be performed safely and lead satisfactory outcomes.

## Highlights

• Intrapancreatic Accessory Spleen (IPAS) is a rare embryological benign abnormality.
• (IPAS) should be considered in patients with recurrent immune thrombocytopenia (ITP).
• Although IPAS is a challenging diagnosis, it can be detected by the recent advancement of medical imaging.

## 1 Introduction

Intra-pancreatic accessory spleen (IPAS) is a benign congenital variance in which there is a failure of fusion between a portion of the splenic tissue and the main body of the spleen during embryologic splenic development. It usually occurs at the fifth week of fetal life and consists of structurally normal splenic tissue [1]. Accessory spleen is present in 10–30% of the population, however, only about 17% of all accessory spleens are located within the pancreatic tail, it is more commonly found in the region of the splenic hilum and vascular pedicle [2]. It is usually asymptomatic and diagnosed as an incidental pancreatic nodule on Computed Tomography (CT) or Magnetic Resonance (MR) scan performed for other purposes [3].

In similar context, immune thrombocytopenia (ITP) is considered the second most common indication for splenectomy after trauma usually indicated after failure of initial medical therapy, provided that the patients can tolerate the surgery and accept the risks. The failure rate of surgery is about 8% and the failure rate after 5 years is around 28%. Its efficacy lies in removing the major site of phagocytosis of antibody-coated platelets and is considered to have the greatest potential to modify the course of disease among the treatment options. [4] IPAS along with ITP can cause recurrent disease after splenectomy and surgery is advised to maintain an acceptable platelet count and moderate treatment [5]. Here, we report a case of 33-year-old male who was diagnosed with chronic ITP and was found to have IPAS that was identified radiologically and removed laparoscopically. The work has been reported in line with SCARE criteria [6].

## 2 Case presentation

The patient is a 33-year-old male with a known case of chronic relapsing ITP diagnosed in 2012. Additionally, he is diagnosed with temporal lobe epilepsy on anti-epileptic medications, Graves’ disease status post radiation of thyroid and on levothyroxine replacement, and valvular heart disease.

**Fig. 1:**
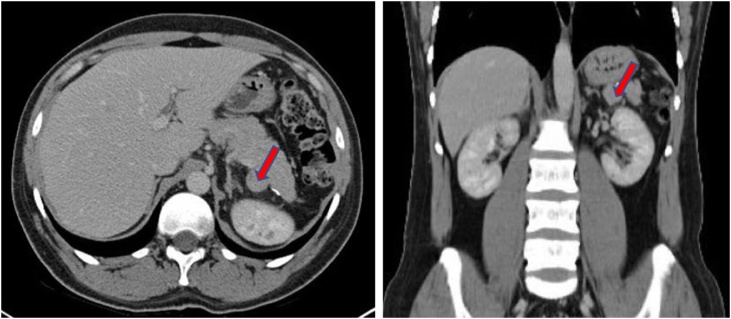
Enhanced CT scan of abdomen showing a well-defined rounded hyperattenuating soft tissue lesion inseparable from the posterior wall of the pancreatic tail measures 2 cm in diameter.

**Fig. 2:**
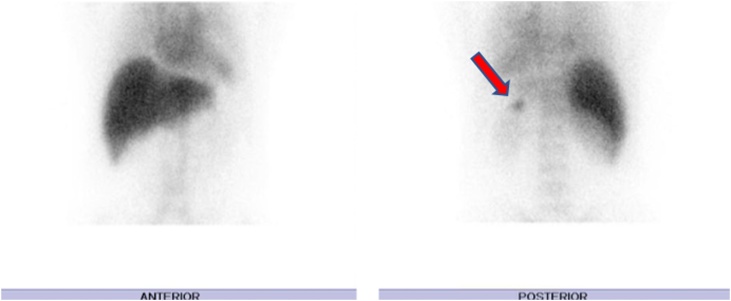
Anterior and posterior images of the abdomen after administration of 925 MBq of technetium99 m sulfur colloid showing accessory spleen within the tail of the pancreas.

The patient presented at the time of diagnosis in April 2012 with severe thrombocytopenia that was complicated by pulmonary hemorrhage which required ICU admission. His investigation at the time showed platelets count of only 1 × 10 ^9^/L (normal range 150–450 × 10^9^/L). The patient was treated with intravenous immunoglobulin (IVIG) and steroids (Prednisone 25 mg/day), responding for 4 months with a rise in platelet count to 62 × 10 ^9^/L. After which he relapsed developing epistaxis, purpura and a platelet count of 2 × 10 ^9^/L. Again, the patient was treated with higher dose of steroid (30 mg/day) and IVIG and a surgical team was consulted for possible splenectomy. A bone marrow aspirate and biopsy was preformed which showed increased megakaryocytes consistent with ITP and was otherwise normal. Laparoscopic splenectomy was done in October 2012, the surgery went well and the patient was discharged in good condition. Three years later in December 2015, he presented again with bleeding and ulcer in the oral cavity and minimal episodes of fresh blood per rectum. He was admitted as a case of chronic relapsing ITP with platelets of 6 × 10 ^9^/L, which increased to 96 × 10 ^9^/L after a short course of steroid (Prednisone 25 mg/day), and IVIG. About 3 months later in March, he relapsed with platelets of 3 × 10 ^9^/L. During hospital course, he underwent CT scan of abdomen and pelvis with contrast which revealed a small-sized mass originating from the medial part of the tail of the pancreas that was suspected to be an accessory spleen (Fig. 1). Thus, a surgical team was consulted and a colloid scan was advised that confirmed accessory spleen originating from the tail of the pancreas (Fig. 2). Repeated bone marrow aspirate and biopsy was done in the same admission to rule out malignancy.

Meanwhile, IVIG and steroid were continued and a laparoscopic accessory splenectomy (LAcS) was performed on the scheduled date in April 2016 as the patient was seen by neurology and cardiology for his conditions and found to be fit for surgery. Platelet count on the day of surgery was 230 × 10 ^9^/L. The intraoperative setting confirmed the radiological data of accessory spleen which was surrounded by a fibrotic capsule that separates it from the adjacent pancreatic parenchyma, consistent with gross examination reported in the literature [5]. Using three trocars including the scope, the lesser sac was opened and pancreas was localized. Then the inferior border of the pancreas was dissected to find the small splenule in the inferior part which was removed from within the pancreas. The surgical time was 80 min, following which there were no post-operative complications. Pathologic assessment of the excised tissue confirmed the specimen to be accessory spleen. As expected, within a few days, platelets increased up to 754 × 10 ^9^/L and the patient was started on Aspirin 81 mg daily for 3 months.

The patient was followed up regularly in hematology clinic and was continued on prednisone 20 mg daily and Eltrombopag 50 mg daily until both were tapered off in May 2016. His platelets continued to range from 265 × 10 ^9^/L to 363 × 10 ^9^/L since then. Although his platelet count dropped slightly during his last clinic visit on October 2018 (30 months’ post-surgery) where it was 130 × 10 ^9^/L, it was still considered acceptable as it did not reach a threshold level that could cause a significant increased risk of bleeding.

## 3 Discussion

Generally, IPAS is an asymptomatic benign lesion and is often discovered incidentally during imaging evaluation for other unrelated diagnoses, which makes surgery not indicated in asymptomatic patients. On the other hand, splenectomy in patients affected with ITP shows a success rate of around 70%. However, 15–30% of patients relapse after surgery and around 30% of these relapses are caused by accessory splenic tissue which are usually readily found close to the lower pole or hilum of the spleen [5,7].

Identifying an accessory spleen becomes especially crucial in patients with ITP, but since IPAS appears radiologically similar to a hypervascular pancreatic lesion it could be easily missed. IPAS tends to be a small, round, and homogeneous lesion with well-defined borders and is usually located within the pancreatic tail. Additionally, it should be noted that IPAS can have a different white-to-red pulp ratio compared to normal spleen, leading to IPAS on T2-weighted imaging to be different from that of the normal spleen which may lead radiologists to make an incorrect diagnosis when not familiar with the histologic-radiologic correlation [8]. Additionally, Tc-99 m sulfur colloid scintigraphy can be utilized as it has also been reported to be effective for differentiating tumors from accessory spleen due to a marked increased focal uptake in functioning splenic tissue that exceeds that of the cardiac blood pool and the major vessels [9].

Since IPAS can cause a recurrence of ITP its removal is recommended when imaging studies conﬁrm its presence. Studies have suggested favorable results of response rates after laparoscopic removal of retained splenic tissues in general [10]. In our experience, we noticed a substantial response of our patient which was in line with the literature for the feasibility of LAcS as a treatment modality.

Woo J. et al, reported less than 25% long-term remission after the removal of an accessory spleen. He attributed his results to the increased destruction of platelets by accessory parts of the reticuloendothelial system other than the spleen [11]. In our study, our long-term effect after LAcS was noticeable as the patient was off steroids for 32 months with satisfactory platelets count.

Tapering of medication dose after accessory splenectomy seems to be the most common beneﬁt according to Szold et al. who reported satisfactory remission after LAcS in eight patients with recurrent ITP [12]. Progress in our patient showed sufficient response after LAcS, which is consistent with most cases described in the literature after the surgical management of ITP recurrence due to accessory spleen.

Although IPAS is a challenging diagnosis, it can be non-invasively detected by an experienced radiologist with the recent advancement of medical imaging. Recognizing IPAS as a differential diagnosis for recurrence of ITP can be safely reached using enhanced CT scan along with Tc-99m sulfur colloid scintigraphy. Furthermore, minimally invasive approach is safe and beneﬁcial to patients suffering from recurrent ITP with documented IPAS resulting in satisfactory outcomes.

## Conflicts of interest

The authors declare no conflict of interest.

## Funding statement

The authors declare no source funding.

## Ethical approval

Approval has been given by ORA at our institute.

## Consent

Written informed consent was obtained from the patient for publication of this case report and accompanying images. A copy of the written consent is available for review by the Editor-in-Chief of this journal on request.

## Author contribution

AlShammari; designing and reviewing the paper.

Kalagi; Literature search and writing manuscript.

Hajji; Literature search and writing manuscript.

AbuRahma; performing surgeon and reviewing article.

## Registration of research studies

Not applicable.

## Guarantor

Abdullah AlShammari.

## Provenance and peer review

Not commissioned, externally peer-reviewed.
